# Supplementary material for: The interactome of intact mitochondria by cross-linking mass spectrometry provides evidence for coexisting respiratory supercomplexes
Source: Mol Cell Proteomics. 2017 Dec 8;17(2):216–32. doi: 10.1074/mcp.RA117.000470 (PMC5795388; doi:10.1074/mcp.RA117.000470)
Supplement: Supplemental Data [file supp_RA117.000470_133922_0_supp_23978_fzffwf.zip › spectra_annotation/mito_spectra_annotation/146-1-15-1-1-1.pdf]

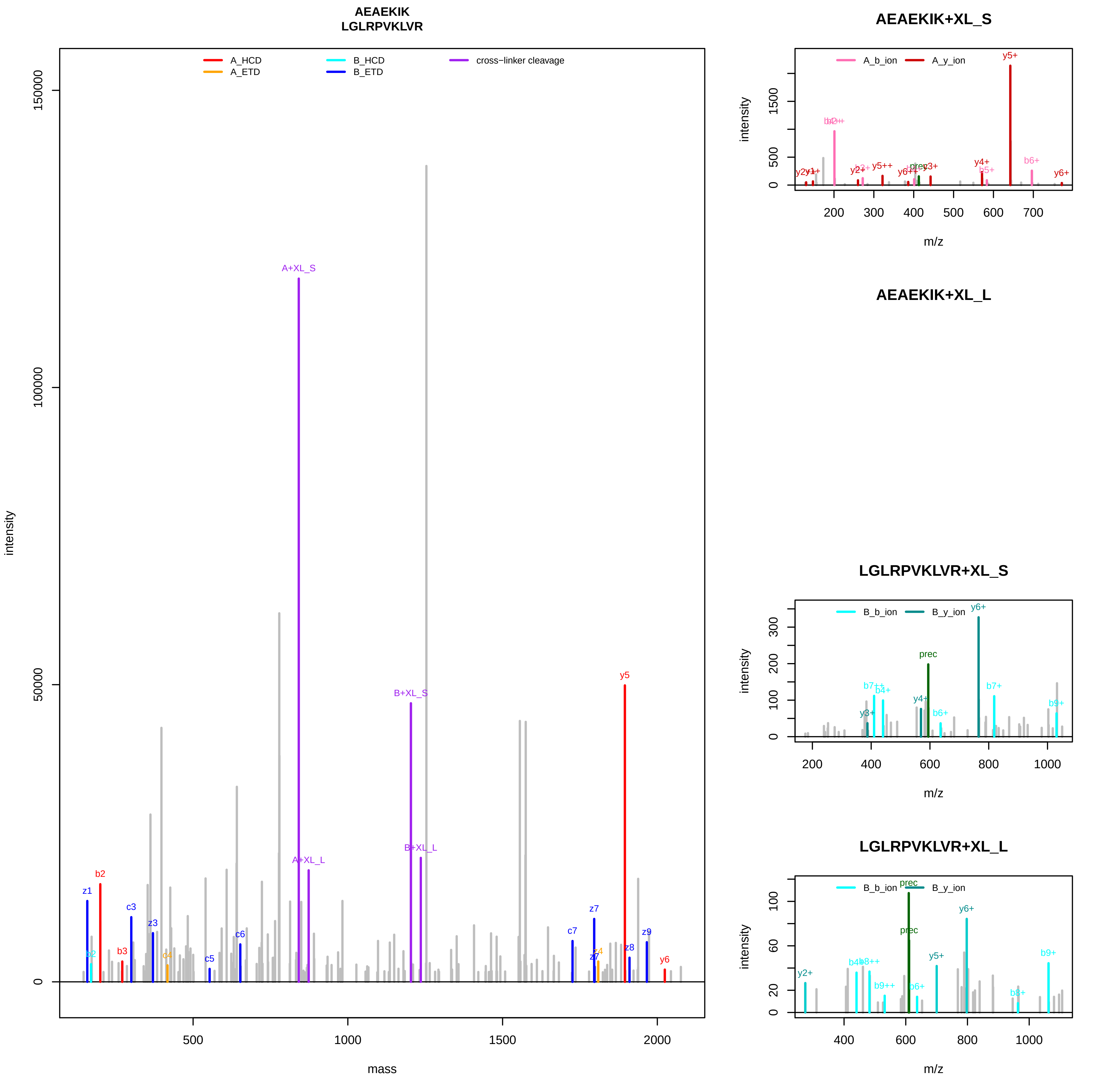
AEAEKIK
LGLRPVKLVR
A_HCD
A_ETD
B_HCD
B_ETD
cross−linker cleavage
intensity
mass
A+XL_S
A+XL_L
B+XL_S
B+XL_L
z1
b2
b3
c3
z3
c4
c5
c6
c7
z7
z4
z8
z9
y5
y6
AEAEKIK+XL_S
A_b_ion
A_y_ion
y5+
b6+
y6+
y4+
b5+
y5++
y2+
y6++
prec
y3+
m/z
AEAEKIK+XL_L
LGLRPVKLVR+XL_S
B_b_ion
B_y_ion
y6+
prec
b7++
b4+
y4+
y3+
b6+
b7+
b9+
LGLRPVKLVR+XL_L
y2+
b4+
b8++
b9++
b6+
y5+
b8+
